# Fabrication of Human Skin Equivalents Using Decellularized Extracellular Matrix

**DOI:** 10.1002/cpz1.393

**Published:** 2022-03-08

**Authors:** Atiya M. Sarmin, John T. Connelly

**Affiliations:** ^1^ Centre for Cell Biology and Cutaneous Research Queen Mary University of London London UK

**Keywords:** biomaterials, extracellular matrix, *in vitro* models, skin, tissue engineering

## Abstract

There is a growing demand for *in vitro* models of human tissues that recapitulate the complex structures and functions found *in vivo*, and the biomaterials that support these physiologically relevant models are essential underpinning technologies. Here, we present an optimized protocol for generating human skin equivalents (HSEs) using a dermal matrix isolated from decellularized porcine skin. The decellularized extracellular matrix (dECM) contains a complex mixture of fibrillar collagens and matrisomal proteins that mimic native skin and can be produced in large quantities. The procedure for decellularization, digestion, and solubilization of the dECM is described in detail. In addition, we provide instructions for how to construct a three‐dimensional HSE model using the dECM as the dermal support matrix for human keratinocytes and dermal fibroblasts. Recent studies from our laboratory have shown that HSEs generated using porcine dECM display improved epidermal differentiation and stratification compared to existing protocols using type I collagen gels. Thus, dECM‐based biomaterials are a useful tool for replicating human skin physiology *in vitro* and developing advanced human skin models for therapeutic discovery and testing. © 2022 The Authors. Current Protocols published by Wiley Periodicals LLC.

**Basic Protocol 1**: Preparation of decellularized extracellular matrix from porcine skin

**Basic Protocol 2**: Generation of human skin equivalents

## INTRODUCTION

Human skin is a complex, multicellular tissue that provides a protective barrier between the body and the external environment. Experimental models that recapitulate this essential function of the skin are critical for understanding normal tissue physiology and disease pathogenesis and the development and testing of therapeutics and consumer health products. Existing *in vitro* models of human skin typically consist of a dermal matrix composed of purified type I collagen and dermal fibroblasts, along with epidermal keratinocytes on the surface. When cultured at the air‐liquid interface, keratinocytes form a stratified epidermis that mimics normal tissue architecture. However, a key limitation of these models is the suboptimal differentiation of the epidermis and incomplete barrier formation.

To improve upon current *in vitro* human skin equivalent (HSE) models, we recently developed a protocol for the decellularization and solubilization of the dermal extracellular matrix (ECM) derived from porcine skin (Sarmin et al., under revision). The decellularized ECM (dECM) retains a complex mixture of fibrillar collagens and matrisomal proteins found within the skin and better mimics the native microenvironment of the tissue. In addition, the dECM forms a stable hydrogel at neutral pH, and when used as a dermal matrix for HSE models, it supports epidermal differentiation and maturation, which is superior to existing type I collagen‐based matrices.

In this report, we provide two detailed protocols for the preparation of dECM from porcine skin and the fabrication of HSEs using dECM as a dermal matrix. Porcine skin was selected as a tissue source as it is highly similar to human skin (Liu et al., 2020) and easily available in large quantities. Decellularization involves a two‐step enzymatic treatment to delaminate the epidermis with Dispase II, followed by removal of dermal cells with Trypsin and a Triton‐based detergent. The ECM proteins are then solubilized by Pepsin digestion under acidic conditions and can be stored frozen for up to 1 year. To generate HSEs, the dECM is neutralized, mixed with human dermal fibroblasts, and gelled at 37°C. Human keratinocytes can then be seeded on the surface of the dECM gel, and when cultured at the air‐liquid interface, a well‐differentiated epidermal layer will form over 14 days. Step‐by‐step instructions for these protocols are listed below.

## Basic Protocol 1 PREPARATION OF DECELLULARIZED EXTRACELLULAR MATRIX FROM PORCINE SKIN

**Figure 1:**
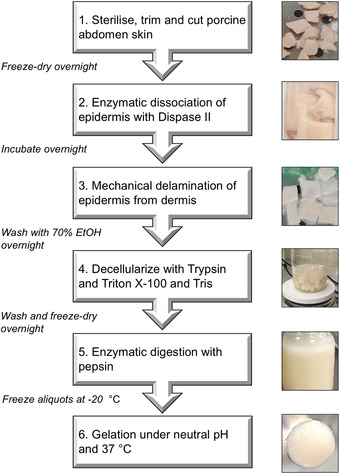
Overview of the porcine skin decellularized ECM protocol. (1) Fresh porcine abdomen skin was trimmed of fat and hair before being cut into 1 × 1 cm pieces. (2‐3) Enzymatic dissociation of the epithelium was performed with Dispase II (50 U/L) followed by mechanical delamination of the epidermis. (4) Decellularization was achieved by trypsinization (0.25%/0.1% EDTA) and washes with a Triton‐X 100 Tris Buffer solution (0.26% EDTA, 1% Triton‐X 100, 0.69% Tris). (5) Enzymatic digestion of the dermis was performed by pepsin (1 mg/ml) digestion to produce tissue at a 20 mg/ml concentration. (6) The dECM hydrogel is gelled once neutralized with one‐tenth of the digest volume of 1 N NaOH and one‐ninth of the digest volume of 10× PBS and incubated at 37°C for 1 hr.

Protocol 1 describes the method for preparing dECM from porcine skin. The procedure begins with 50‐100 g of skin from pig belly, which can be obtained from a local butcher or abattoir, and it requires 7‐10 days to complete. An overall summary of the workflow is shown in Figure 1, and it is recommended that the entire protocol is reviewed carefully before beginning. Decellularization involves removal of the epidermis with the enzyme Dispase, followed by combined Trypsin and detergent treatment to remove the cells of the dermis. The decellularized dermal tissue is then freeze‐dried and solubilized with Pepsin to yield a 20 mg/ml solution of dECM. The dECM can be portioned into small aliquots and stored at −20°C for up to 1 year. Note: an initial starting mass of 50‐100 g (20 × 10 cm) of porcine skin can be expected to produce approximately 500 ml of soluble dECM.

### Materials

Pig skin (Local butcher, pork belly skin, 50‐100 g)
PBS (ThermoFisher, P4417)
Penicillin‐Streptomycin (Pen/Strep, ThermoFisher, 15140122)
NaAc (Sigma‐Aldrich, S8750)
CaCl_2_ (Sigma‐Aldrich, 902179)
Dispase II (Merck, D4693‐1G, see recipe)
Trypsin (ThermoFisher, 25200056)
Pepsin A (Sigma‐Aldrich, P7000)
Triton X‐100 (Sigma‐Aldrich, T8787)
EDTA (Sigma‐Aldrich, 1001429355)
Tris (Sigma‐Aldrich, T1503)
Concentrated HCl (Sigma‐Aldrich, 32033)
Sterile water (MilliQ or equivalent)
Ethanol (VWR, 8.18760.2500 or equivalent)

Balance (Mettler‐Toledo, AG204 DeltaRange)
−80°C freezer (Eppendorf, CryoCube F570)
−20°C freezer (ThermoFisher, GGU 1500, 12048281)
4°C fridge (Liebherr, GKV4310)
Lyophilizer (Edwards, Modulyo)
Scalpel (Swann Morton, 0501)
Tweezers (SLS, INS4292)
Magnetic stir bars, sterile (SLS, STI5018)
50‐ml centrifuge tubes (ThermoFisher, Nunc, 339652)
Tube roller (Stuart, SRT9D)
Stirrer/hot plate (ThermoFisher, Isotemp RT Advanced, 15423692
Sterile filter (ThermoFisher, Stericup Vacuum Filter, S2GPU01RE)
Parafilm (SLS, FIL1020)
Glass bottle (Corning, Pyrex, 500 ml)

### Day 1

1 In a biological safety cabinet, rinse the fresh porcine skin with 70% ethanol and PBS. All subsequent steps should be carried out under sterile conditions
2 Cut the skin into small pieces of approximately 1 × 1 cm using scalpels, and remove as much fat and hair as possible.
  Change scalpels once each of them becomes dull. Surgical scissors can also be used.
3 Soak the skin in PBS supplemented with 1% Pen/Strep for 5 min.
4 Pour away the dPBS/PenStrep solution and transfer the skin pieces into 50‐ml centrifuge tubes.
5 Weigh the skin in each tube and aim for approximately 25‐40 g (100 pieces) per tube.
  Record the empty tube weight to assist with measuring the dry weight on day 2.
6 Freeze the skin at −80°C until completely frozen (approximately 4‐5 hr or overnight).
7 Freeze‐dry the skin overnight within the lyophilizer with the caps of the tubes loosened or covered with parafilm with holes in it.
  Efficient removal of the water by sublimation in the lyophilizer requires the tissue to be completely frozen beforehand (step 6).

### Day 2

8 Reweigh the freeze‐dried tubes and determine the dry tissue mass.
  Dry tissue mass = [Mass of the tube with dried tissue] – [Mass of the empty tube from step 5]
9 Prepare the Dispase II solution at a final concentration of 560 U/L and make enough for 2 ml of Dispase solution per gram of dry skin.
10 To the same tubes, add 2 ml of Dispase solution for every gram of freeze‐dried skin. Do not exceed 40 ml in each tube to allow space for stirring.
11 Seal the tubes and place them on a roller, similar to the one listed above, on medium speed at 4°C overnight.

### Day 3

12 After approximately 12‐16 hr incubation in Dispase, transfer the skin to a sterile petri dish in a biological safety cabinet. Gently scrape off the epidermis from each piece of skin with tweezers.
13 Wash the dermis three times with sterile water.
14 Wash the dermis again with 70% ethanol in a sterile 500‐ml glass bottle overnight at room temperature with continuous mixing on a stir plate.

### Day 4

15 Transfer the dermis to a clean, sterile bottle. Cover the dermis with 0.25% trypsin and incubate for 1 hr on a 40°C hot plate with stirring.
  Alternatively, a 37°C incubator can be used instead of the hot plate.
16 Wash the dermis three times with sterile water and pour off the excess.
17 Prepare a decellularization solution of 1% Triton‐X‐100, 0.25% EDTA, and 0.69% Tris (see recipe). Add enough of the solution to cover the tissue and incubate for 6 hr at room temperature with continuous stirring.
18 Replace the solution with fresh Triton/EDTA/Tris solution and incubate overnight at room temperature with stirring.

### Day 5

19 Wash the dermis three times with sterile water.
20 Transfer the dermis to new sterile 50‐ml centrifuge tubes and freeze at −80°C.
  This step can be over the weekend. Note the weight of the empty tubes for calculating the dry tissue mass.

### Day 6

21 Freeze‐dry the tissue overnight in the lyophilizer with loosened caps or cover with parafilm with poked holes.
  As noted above, ensure the tissue is completely frozen before the freeze‐drying process

### Day 7

22 Prepare pepsin solubilization solution by dissolving 600 mg Pepsin in 600 ml of 0.01 M HCl. Filter sterilize.
23 Reweigh the tubes and determine the mass of dry tissue. Transfer the dry tissue to a sterile glass bottle and add 1 ml of the Pepsin solution for every 20 mg of freeze‐dried tissue.
  This step will result in a final dECM concentration of 20 mg/ml, which we find works well for HSEs, but the concentration can be adjusted based on the final application.
24 Incubate at room temperature on a magnetic stirrer for 3 days or until all tissue is fully dissolved.
25 Prepare 10 ml aliquots of the dECM and store them at −20°C for up to 1 year.

## Basic Protocol 2 CONSTRUCTION OF HSEs USING dECM AS A DERMAL MATRIX

This protocol describes how to construct three‐dimensional (3D) HSEs using the dECM materials as the dermal support matrix. The procedure involves embedding human dermal fibroblasts in neutralized dECM, forming a soft gel at 37°C. Human keratinocytes are then seeded on the surface and cultured at the air‐liquid interface to form a stratified and differentiated epidermal layer over 14 days. We recommend using the dECM at a concentration of 20 mg/ml, but this can be adjusted to modify the density or stiffness of the dermal matrix as desired.

### Materials

Frozen aliquot of dECM (see Basic Protocol 1)
10 × PBS (ThermoFisher, 70011044)
NaOH (Sigma Aldrich, S5881)
FAD medium (see recipe)
Ascorbic acid (Sigma Aldrich, A92902)
Primary human dermal fibroblasts (isolated from neonatal skin) (Rikken, Niehues, & van den Bogaard, 2020)
Human keratinocytes (primary isolated from neonatal or adult skin or immortalized lines) (Rikken, Niehues, & van den Bogaard, 2020)

12‐well plates (ThermoFisher, Nunc 150628)
12‐well transwell inserts, 0.4‐mm PTFE (Merck Millipore, MCHT12H48)
Cloning rings (Bel‐Art, 378470300)
Cell culture incubator, 37°C, 5% CO_2_ (ThermoFisher, Heracell, 11666310)

### Preparing the gel‐fibroblast mix

1 Thaw the required volume of dECM at 4°C for 3‐4 hr and place on ice until use. Prepare enough dECM for 340 μl per HSE plus 10% excess for wastage.
2 Prepare a cell suspension of primary human dermal fibroblast cells at 1 × 10^6^ cells/ml in DMEM medium supplemented with 10% FBS and 1% pen/strep. Prepare enough cells for 3.4 × 10^4^ fibroblasts per HSE.
3 Combine the dECM with one‐tenth the final volume of 1N NaOH and one‐ninth the final volume of 10× PBS, then add in one‐tenth the final volume of the cell suspension.
  Mix gently with a 1‐ml pipette by stirring WITHOUT pipetting up and down to prevent bubbles.
  The final fibroblast density will be 1 × 10^5^ cells/ml.

### Casting the fibroblast embedded dECM gels

4 Place Transwell inserts in each well of the 12‐well plate.
5 Add 340 μl of the gel and fibroblast mix to the center of each Transwell insert.
6 Incubate in a sterile 37°C incubator for at least 1 hr to set. Keratinocyte can be added immediately after the gel is set or up to 24 hr later.
7 After the gel has set, gently insert a cloning ring with the larger opening facing up and the smaller opening on the gel side.

### Seeding keratinocytes onto the dECM surface

8 Prepare a cell suspension of human keratinocytes in complete FAD medium at a density of 1 × 10^6^ cells/ml.
  The seeding density may need to be optimized for different keratinocyte lines, but 1 × 10^6^ cells/ml is recommended for primary keratinocytes and the NTERT keratinocyte line (Dickson et al., 2000).
9 Into the center of each gel enclosed within the cloning ring, add 100 μl of the cell suspension drop by drop.
  The cloning ring helps keep the keratinocytes on the surface of the gel and prevents the cell suspension from running down the side.
10 To the cell suspension, add 100 μl of FAD medium.
11 Add 500 μl of FAD medium to the bottom of the well. Add the medium slowly down the side of the well to avoid generating bubbles.
  This should be enough to cover the bottom of the insert but not go over the sides into the middle of the insert. At this point, there will be 200 μl in the insert and 500 μl in the lower well, 700 μl in total.
12 Place the plate in a 37°C incubator overnight.

### Raising HSE cultures to the air‐liquid interface

13 After 24 hr of submerged culture, the HSEs are raised to the air‐liquid interface. First, carefully remove the cloning ring from each HSE with sterile tweezers.
14 Gently remove the medium from the insert by pipetting and replace the medium in the bottom well with 500 μl of fresh FAD medium. The air‐liquid interface is created by leaving the top of the insert exposed to the air, while the bottom remains in contact with the medium in the well through the insert membrane.
15 Culture the gels at 37°C and 5% CO_2_ for 14 days, replacing the medium in the bottom well every day.
16 Starting on day 8 of the culture, begin supplementing the FAD medium with 50 μg/ml of fresh ascorbic acid (vitamin C). This step encourages collagen deposition and stabilization of the dermal matrix in the later stages of the culture period.
17 At the end of the culture period, HSEs can be fixed with paraformaldehyde, paraffin‐embedded, and processed for histology and immunofluorescence using standard protocols (Schacht & Kern, 2015).
  The HSEs will be more fragile than normal skin and should be handled gently. They can be removed from the insert by cutting out the membrane with a scalpel and carefully peeling the membrane off the HSE.

## REAGENTS AND SOLUTIONS

*Use deionized, distilled water in all recipes and protocol steps*.

### Dispase II solution

A 50 U/ml stock Dispase solution should first be prepared by dissolving the powder in a buffer containing 10 mM NaAc (pH 7.5) and 5mM CaCl2.
Dilute the stock to 560 U/L in water and use fresh.

### FAD medium

Prepare a base medium of 250 ml DMEM (ThermoFisher, 31966021) and 250 ml DMEM/F12 (50/50, ThermoFisher, 11320033), 10% (v/v) FBS (Biosera, FB‐1285), and 1% (v/v) Pen/Step (ThermoFisher, 15140122).
Supplement with 10^−10^ M cholera toxin (Sigma Aldrich, C8052), 0.5 μg/ml hydrocortisone (ThermoFisher, 352450010), 10 ng/ml EGF (Peprotech, 100‐15), and 50 μg/ml insulin (Sigma Aldrich, I5500). The complete FAD medium can be stored at 4°C for up to 1 month.

### Pepsin solubilization solution

Prepare 600 ml of 0.01 M HCl (diluted in water from concentrated stock) and dissolve 600 mg Pepsin in the HCl solution. Filter sterilize. Use fresh.

### Trypsin/Triton decellularization solution

Prepare a solution of 1% (v/v) Triton‐X‐100, 0.25% (w/v) EDTA, and 0.69% (w/v) Tris in water. Use fresh.

## COMMENTARY

### Background Information

Tissue‐engineered human skin equivalents (HSEs) have been developed to recapitulate key structural and functional features of native skin (MacNeil, 2007; Reijnders et al., 2015; Zhao et al., 2016). They are a common alternative to traditional two‐dimensional cell culture and animal models and typically consist of an ECM‐mimetic dermal scaffold laden with fibroblasts and covered with a stratified layer of keratinocytes. While these bi‐layered models replicate the basic structure of the skin, the barrier function is still less well‐developed than native tissue (Randall, Jüngel, Rimann, & Wuertz‐Kozak, 2018; Schmidt, Nowakowski, & Kluger, 2020), and there is a lack of more complex hair follicles and glandular structures. Recent efforts to produce *in vitro* HSEs that more closely resemble human skin aim to incorporate additional features such as a hypodermis (Bellas, Seiberg, Garlick, & Kaplan, 2012), vasculature (Groeber et al., 2016), or immune cells (Lorthois, Simard, Morin, & Pouliot, 2019), and make use of advanced biofabrication methods such as 3D bioprinting (Kim, Lee, Gao, & Cho, 2017; W. Lee et al., 2009; Min et al., 2018).

As the ECM provides essential physical and biochemical signals for regulating skin structure and function, identifying appropriate biomaterials for mimicking the dermal matrix is a key consideration in HSE development. While previous studies have investigated a wide range of natural polymer hydrogels, such as fibrin (Mazlyzam et al., 2007), type I collagen (Lee et al., 2009; Vidal et al., 2019), Matrigel (Kleinman & Martin, 2005; Shamir & Ewald, 2014), and gelatin (Pourchet et al., 2017), more recent research has begun to explore the use of decellularized ECM (dECM) derived directly from the skin (Choi et al., 2011; Choi et al., 2012; H. Lee et al., 2016). Decellularization is achieved by removing cells and cellular remnants with detergents while minimizing loss and damage of the native ECM and retaining its composition and ultrastructure as much as possible (Xing et al., 2015). Decellularized tissues can also be solubilized by protease treatment and fabricated into stable hydrogels (Coronado et al., 2017). It is believed that dECM hydrogels are advantageous as they retain the biochemical complexity, biological activity, and nanostructure of the endogenous tissue.

Despite these advantages, there are still some notable limitations of dECM hydrogels. Their mechanical properties are often inferior to the native tissue and do not display the same level of strength and stiffness. Unlike synthetic polymers, dECM hydrogels also have limited tuneability in terms of density/stiffness, chemical composition, and nanostructure. However, blending the dECM with other biomaterials may offer a route to achieving optimal biological and mechanical properties. Another potential disadvantage of using animal‐derived dECM, as we describe here, is a mismatch between human cells and ECM from another species. While we have not observed any negative impacts of porcine ECM on human fibroblasts and keratinocytes to date, there is still the possibility of immunogenic reactions if immune cells are introduced to the HSE models or if dECM materials are used for transplantation and tissue repair.

### Critical Parameters

A number of factors are essential for the successful generation of HSE models using dECM materials. During dECM preparation, it is important that the ECM is completely freeze‐dried prior to decellularization and prior to solubilization. This step requires that the tissue is first completely frozen and that the lyophilization system performs well (maintains low pressure and temperature). The other critical step for the preparation of the dECM is ensuring complete digestion of the ECM with pepsin, as this will result in a more homogeneous and consistent dermal matrix for the HSE model.

When constructing the HSE model, two key steps are carefully casting the dECM gel and ensuring that the culture remains at the air‐liquid interface. Care should be taken to avoid generating bubbles within the dECM gel and pipetting uniform and equal volumes within the transwell insert. Likewise, throughout the 14‐day culture period, care should be taken to keep the epidermal layer at the air‐liquid interface by not getting medium on the surface of the HSE and making sure that the bottom of the transwell insert makes uniform contact with the medium in the lower well.

### Troubleshooting: (Table 1)

**Table 1.** Troubleshooting Guide for Generation of HSE Models Using dECM Supports

Problem	Possible Cause	Solution
Incomplete pepsin digestion	Lower enzyme activity	Extend the digestion until no solid pieces of tissue are visible
dECM gels do not solidify after 1 hr	The concentration of the dECM is too low or pH is not neutral	Allow gels to set for an additional hour. If still not gelled, check the pH or try preparing a more concentrated dECM
Poor epidermal stratification and differentiation	Cultures not at the air‐liquid interface	Check the volumes of medium in the bottom of the well. Alternative brands of well plates and inserts may require adjusted volumes of medium

### Understanding Results

**Figure 2:**
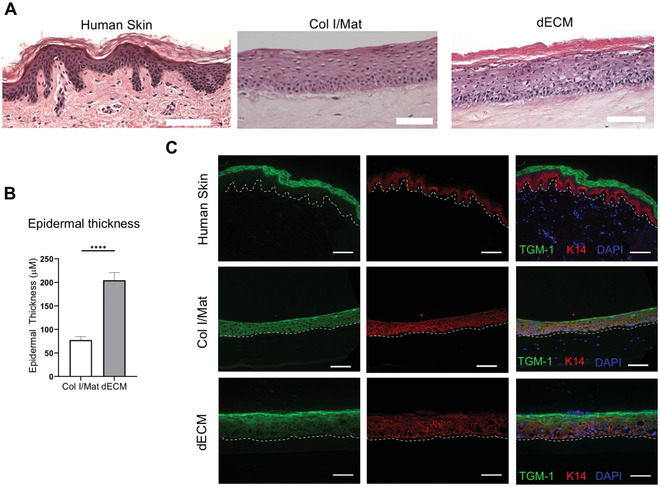
Construction of HSE models using dECM hydrogels. (A) H&E staining of human skin, 3D HSEs constructed with type I collagen/Matrigel (Col I/Mat) or dECM gels. Scale bars represent 200 μm. (B) Quantification of epidermal thickness for Col I/Mat and dECM HSEs. Data represent mean ± SEM of *N* = 3 experiments. (C) Immunofluorescence images of transglutaminase 1 (TGM‐1) and keratin 14 (K14) in human skin and HSEs constructed with Col I/Mat or dECM gels. Scale bars represent 200 μm. The dashed line indicates the dermal‐epidermal junction.

When used as a dermal matrix for HSEs, the dECM should produce a skin model with a well‐differentiated epidermal layer. There should be a clear cornified layer with anuclear cells visible by H&E staining, and the overall thickness of the epidermis should be greater than that of the epidermis generated with a collagen/Matrigel dermal matrix using standard protocols (Enjalbert et al., 2020) (Figs. 2A,B). Likewise, there should be a clear distinction between the proliferative basal keratinocytes expressing keratin 14 and terminally differentiated keratinocytes in the upper layers, marked by proteins such as involucrin, loricrin, or transglutaminase (Fig. 2C). In contrast to human skin, keratin 14 expression can often be observed in the suprabasal layers of HSEs, particularly if using immortalized cell lines, and this limitation is a potential area for future improvements. Finally, the dermal layer should appear as a dense fibrous ECM, which becomes progressively contracted over the 14‐day culture period (Fig. 2A).

### Time Considerations

Preparation of the dECM takes approximately 2 weeks in total, and some steps (e.g., freezing) can be performed overnight or over the weekend. Construction of the HSE model requires one full day followed by 2 weeks of culture prior to fixation and analysis.

### Author Contributions

**Atiya Sarmin**: Investigation, methodology; **John Connelly**: Conceptualization, supervision, original draft, review, and editing.

### Conflict of Interest

The authors declare no conflicts of interest.

## Data Availability

Data will be made available upon request to the authors.
